# Treatment Duration in Bacterial Prosthetic Joint Infections: A Narrative Review of Current Evidence

**DOI:** 10.3390/antibiotics14111066

**Published:** 2025-10-25

**Authors:** Hajer Harrabi, Christel Mamona-Kilu, Eloïse Meyer, Emma d’Anglejan Chatillon, Nathalie Dournon, Frédérique Bouchand, Clara Duran, Véronique Perronne, Karim Jaffal, Aurélien Dinh

**Affiliations:** Infectious Disease Department, Raymond-Poincaré University Hospital, APHP Paris Saclay, 92380 Garches, France; hajer.harrabi@aphp.fr (H.H.); christel.mamonakilu@aphp.fr (C.M.-K.); eloise.meyer@aphp.fr (E.M.); emma.danglejanchatillon@aphp.fr (E.d.C.); nathalie.dournon@aphp.fr (N.D.); fbouchand@lesabondances.fr (F.B.); clara.duran@aphp.fr (C.D.); veronique.perronne@aphp.fr (V.P.); karim.jaffal@aphp.fr (K.J.)

**Keywords:** PJI, treatment, antibiotic duration, DAIR, surgery, one-stage exchange, two-stage exchange, arthroplasty

## Abstract

**Background/Objectives:** The optimal duration of antibiotic therapy for bacterial prosthetic joint infections (PJI) remains a topic of considerable debate. Current recommendations are often based on limited evidence and expert consensus. Emerging data suggest that shorter antibiotic courses may be as effective as prolonged treatments in select cases. Shortening the duration of therapy offers several advantages, including a reduced risk of bacterial resistance, fewer adverse events, and cost savings. However, this approach must be carefully balanced with the individual patient’s risk of treatment failure. This narrative review aims to synthesize current evidence regarding the duration of antibiotic therapy in PJIs, according to surgical strategies—DAIR (debridement, antibiotics, and implant retention), one-stage exchange, two-stage exchange, and resection without reimplantation—and to identify parameters that may guide individualized and potentially shortened regimens. **Methods:** We conducted a comprehensive search of PubMed, Embase, and Cochrane Library databases through January 2025, including observational studies, randomized controlled trials, and international guidelines. Reference lists of key articles were also screened. **Results:** Studies on DAIR suggest that longer regimens (e.g., 8–12 weeks) are necessary, especially in staphylococcal infections, as confirmed by the DATIPO trial, which showed higher failure rates with 6 weeks compared to 12 weeks. Evidence on one-stage exchange is limited but increasingly suggests that 6 weeks may be sufficient in selected patients; however, no dedicated trial has confirmed this. In two-stage exchange, small retrospective series report successful outcomes with short antibiotic therapy combined with local antibiotics, but randomized trials show trends favoring longer regimens. For patients treated with permanent resection arthroplasty, arthrodesis, or amputation, antibiotic durations are highly variable, with few robust data. Across all strategies, most studies are limited by methodological weaknesses, including small sample sizes, retrospective design, lack of microbiological stratification, and heterogeneous outcome definitions. **Conclusions:** Despite growing interest in shortening antibiotic durations in PJIs, high-quality evidence remains limited. Until additional randomized trials are available—particularly in one- and two-stage exchange settings—12 weeks remains the safest reference duration for most patients, especially those with retained hardware. Future studies should incorporate stratification by infection type, causative organism, and host factors to define tailored and evidence-based antibiotic strategies.

## 1. Introduction

Prosthetic joint infections (PJI) represent a severe and complex complication in orthopedic surgery, significantly affecting patient morbidity and placing a considerable burden on healthcare systems [1]. The 15-year incidence of bacterial PJI is estimated to be in the range of 1.5% to 2%, with a 5-year mortality rate exceeding 20% [1,2]. In 2017, the incidence of hip and knee PJI in the United States was reported at 2.1% and 2.3%, respectively, with similar rates observed in Korea [1]. In France, PJIs account for approximately 4% of all osteo-articular infections [3,4]. By 2030, hospital costs related to hip and knee PJI in the United States are expected to reach $1.85 billion annually [1].

PJI are foreign-body-associated infections that necessitate both medical and surgical management, typically involving the removal of the prosthetic implant and prolonged antibiotic therapy [1,2,4,5,6]. The choice of surgical procedure depends on factors such as the type of infection (acute vs. chronic), the microorganism involved, and the patient’s comorbidities. In cases of chronic infection, the preferred approach is the removal of the arthroplasty, usually followed by the implantation of a new prosthetic device in a one- or two-stage exchange procedure [1,4,5,6]. For patients with acute PJI, a more conservative surgical option, known as Debridement Antibiotics and Implant Retention (DAIR), could be performed [1,4,5,6]. According to the 2013 Infectious Diseases Society of America (IDSA) guidelines, the duration of antibiotic treatment for all type of PJIs ranges from 3 to 6 months, primarily depending on the site of infection [6]. In 2018, the International Meeting on Musculoskeletal Infections stated that the optimal length of antibiotic treatment following DAIR remains uncertain due to considerable variability in the duration, dosage, and administration of treatment [7]. However, it is recommended that the duration of antibiotic therapy for PJI managed with DAIR should be no less than 6 weeks [7,8,9]. These extended durations are recommended mainly due to the presence of biofilm-embedded bacteria. However, the duration of antibiotic therapy remains a topic of debate, with short-course treatments offering several advantages, such as limiting the emergence of bacterial resistance, reducing adverse events, and lowering costs [10].

In this narrative review, we aim to synthesize available data on antibiotic treatment duration in bacterial PJI. Additionally, we seek to identify criteria that can help clinicians safely reduce the duration of treatment without compromising patient outcomes

## 2. Methods/Search Strategy

**Figure A1:**
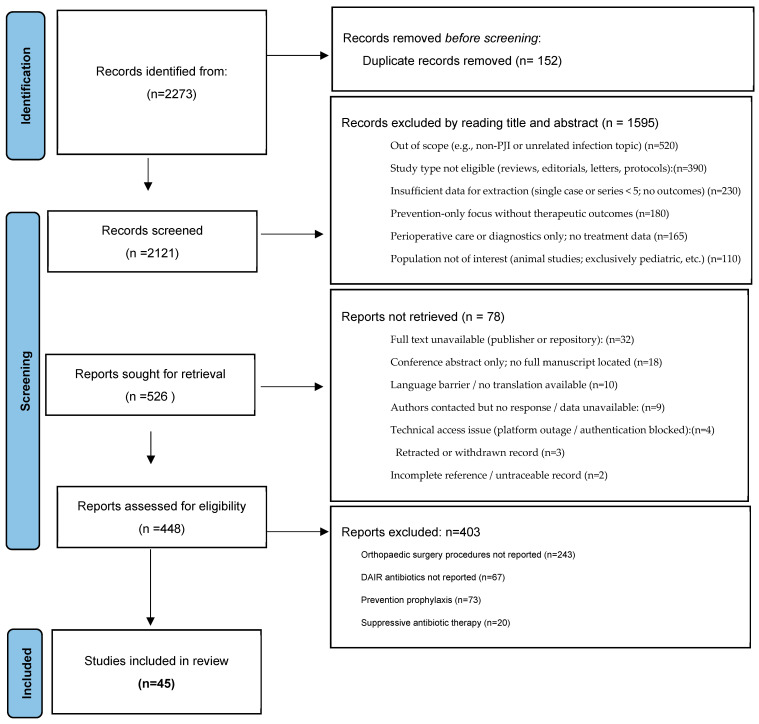
PRISMA flowchart of the literature review.

A comprehensive literature search was conducted using PubMed, Embase, and Cochrane Library databases for publications or abstracts in the English language published up to January 2025. Keywords included “Prosthetic Joint Infection”, “Antibiotic Therapy”, “Treatment Duration”, and “Surgical Procedures”. Additional references were identified from the reference lists of relevant articles. We identified 2305 records; after removing 152 duplicates, 2121 records were screened, with 1595 excluded on title/abstract. Of 526 reports sought, 78 were not retrieved; 448 full texts were assessed and 403 excluded (orthopaedic procedures, *n* = 243; DAIR without antibiotic data, *n* = 67; prophylaxis/prevention, *n* = 73; suppressive therapy, *n* = 20). Forty-five studies were included in this narrative review (Figure A1).

## 3. General Considerations

Accurate diagnosis of PJI is a challenging issue and requires a combination of clinical signs, microbiological evidence, and imaging findings. Definitions differed depending on the Societies/Medical associations and clinicians [6,7,8,9]. Moreover, various classification systems exist according to onset of symptoms, pathogenesis and clinical manifestations: acute, often hematogenous in origin, versus late chronic [11,12,13].

These classifications could help to determine the optimal surgical technique, possibly the appropriate duration of antibiotic therapy, and the prognosis of PJI [11,12].

A collaborative approach involving orthopedic surgeons, infectious disease specialists, radiologists, microbiologists, pathologists, pharmacists and patients is essential in managing PJI. Treatment typically combines surgical intervention with systematic antibiotic therapy. Surgery remains the major pillar in the management of PJI. Surgical treatment options include DAIR (debridement, antibiotics and implant retention), one-stage or two-stage exchange, resection arthroplasty, arthrodesis, and amputation [1,5,6]. Among these, the surgical treatment of reference is complete removal of the arthroplasty [1,5,6].

In this report, we discuss the duration of antibiotic therapy in bacterial PJI according to the type of surgical procedure performed. We considered this approach even if the choice of a surgical procedure is often driven by the surgeon’s preference derived more by empirical experience than evidence-based medicine.

## 4. Antibiotic Duration of PJI According to the Surgery Options

### 4.1. Prosthetic Joint Infection Treated with DAIR (Table 1 and Table 2)

DAIR remains the most commonly used surgical strategy for the management of PJI and is particularly favored in early infections or hematogenous cases with a stable implant and a short symptom duration. The recommended antibiotic duration after DAIR is about 3 to 6 months according to US guidelines [6].

**Table 1:** Summary of Non-Comparative Studies on Antibiotic Treatment Duration in Prosthetic Joint Infections Managed with DAIR.

Study and Year	Design and Sample Size	Dominant Organisms	Therapeutic Approach (Systemic)	Treatment Exposure (Mean)	Follow-Up (Months)	Clinical Success (%)
Berdal 2005 [14]	Prospective; *n* = 29	*Staphylococcus aureus*	Rifampicin + ciprofloxacin	≈3 months (total)	22.5	83
Soriano 2006 [15]	Prospective; *n* = 39	Gram-positive cocci	Levofloxacin + rifampicin	2.7 ± 1 months (total)	24	76.6
Martinez-Pastor 2009 [16]	Prospective; *n* = 47	Enterobacteriaceae	IV β-lactam → oral fluoroquinolone	IV 14 days; oral 2.6 months	15.4	74.5
Cobo 2011 [17]	Prospective; *n* = 117	Gram-negative strains; Gram-positive cocci	Not specified	≈2.5 months (total)	25	57.3
Vilchez 2011 [18]	Prospective; *n* = 53	*Staphylococcus aureus*	IV → oral sequence (details in text)	IV 11 ± 7 days; oral 88 ± 46 days	24	75.5
Tornero 2016 [19]	Prospective; *n* = 143	Gram-negative; Gram-positive cocci	Fluoroquinolone-based; rifampicin combinations	IV 8 days; oral 69 days	48	88.2

**Table 2:** Summary of comparative Studies on Antibiotic Treatment Duration for Prosthetic Joint Infections managed by DAIR.

Study and Year (Ref.)	Design and Setting	Population/Arms	Dominant Organisms	Principal Regimens	Exposure Window	Outcomes and Conclusion
Bernard 2010 [3]	Prospective, observational, single-center	*n* = 144 episodes; 6-week arm (*n* = 70) vs. 12-week arm (*n* = 74)	Staphylococci (~66%)	Rifampicin-based combinations common; also ciprofloxacin, vancomycin, amoxicillin–clavulanate	6 weeks vs. 12 weeks (systemic)	Overall cure 80% (115/144); by arm: 90% with 6 weeks vs. 55% with 12 weeks; authors suggest 6 weeks may suffice; RCTs needed.
Puhto 2012 [20]	Retrospective, pre–post, single-center	ITT: long *n* = 60, short *n* = 72; PP: long *n* = 38, short *n* = 48	*Staphylococcus aureus* (~42%)	Gram-positive regimens mainly rifampicin + fluoroquinolone	Short 2–3 mo vs. long 3–6 mo	Non-inferiority of short therapy: ITT cure 57% vs. 58% (*p* = 0.85); PP 89% vs. 87% (*p* = 0.78). Short course appears acceptable; randomized data urged.
Lora-Tamayo 2013 [21]	Retrospective, multicenter	Total *n* = 231 stratified by duration: <61 d (*n* = 52), 61–90 d (*n* = 52), >90 d (*n* = 127)	*Staphylococcus aureus*; rifampicin use > 75%	Predominantly rifampicin-based combinations	Three strata: <61 d; 61–90 d; >90 d	Cure rates similar across strata: 75%, 77%, 77% (*p* = 0.434). Longer exposure did not improve outcomes.
Lora-Tamayo 2016 [22]	Randomized, open-label, multicenter clinical trial	*n* = 63; ITT long *n* = 33 vs. short *n* = 30; PP long *n* = 20 vs. short *n* = 24	Staphylococci	Levofloxacin + rifampicin (L + R)	Short 8 weeks vs. longer standard (≈3 mo hip; 6 mo knee)	Non-inferiority signal: ITT cure 58% long vs. 73% short (Δ −15.7%, 95% CI −39.2 to +7.8); PP 95% vs. 92% (Δ +3.3%, 95% CI −11.7 to +18.3). Eight weeks L + R may be adequate in DAIR-managed acute staphylococcal PJI.
Chaussade 2017 [23]	Retrospective, multicenter	*n* = 87; 6-week arm *n* = 44; 12-week arm *n* = 43	Staphylococci (~40%)	Rifampicin-based for Gram-positive; fluoroquinolones frequently used	6 weeks vs. 12 weeks	Cure: 70.5% (short) vs. 67.4% (long); adjusted OR 0.76 (95% CI 0.27–2.10). No advantage for 12 weeks; prospective RCTs recommended.
Bernard 2021 (DATIPO) [4]	Randomized, open-label, multicenter	*n* = 151; 6-week *n* = 75; 12-week *n* = 76	*Staphylococcus aureus* ~30–40%	Rifampicin-based combinations; fluoroquinolones commonly paired	6 weeks vs. 12 weeks	Failure: 30.7% (6 weeks) vs. 14.5% (12 weeks); difference 16.2% (95% CI 2.9–29.5). Non-inferiority of 6 weeks not demonstrated.

Notes: Exposure window reflects total systemic duration (short vs. long) as defined by each study. Outcome metrics and cure/failure definitions follow the original authors. DAIR—debridement, antibiotics, and implant retention. ITT: intention-to-treat analysis. PP: per-protocol analysis. 95% CI: 95% confidence interval.

Several studies, both observational and randomized, have investigated the optimal duration of antibiotic therapy following DAIR for PJI, with heterogeneous findings influenced by pathogen type and infection chronicity.

Early non-comparative prospective cohorts [14,15,16,17,18,19] reported treatment durations ranging from 2.5 to 3 months, combining short intravenous (IV) courses (8–14 days) followed by oral therapy, and success rates varying from 57.3% (Cobo et al., predominantly Gram-negative pathogens [17]) to 88.2% (Tornero et al., mostly Gram-positive cocci treated with fluoroquinolone-rifampicin combinations [19]) (see Table 1). These studies lacked stratification by infection stage or pathogen, but suggest better outcomes in acute staphylococcal PJI treated with rifampicin-based regimens.

Comparative studies have yielded mixed results. In a large multicenter retrospective study, patients with *S. aureus* PJI managed with DAIR had similar cure rates whether treated for <61 days (75%), 61–90 days (77%), or >90 days (77%) (*p* = 0.434), suggesting limited benefit of prolonged therapy over 2 months in acute settings [22] (see Table 2). Similarly, Puhto et al. found no difference between short (2–3 months) and long (3–6 months) antibiotic courses in a cohort where 42% of isolates were *S. aureus*. Intention-to-treat (ITT) and per-protocol (PP) cure rates were 58% vs. 57%, and 87% vs. 89%, respectively [20]. In the only randomized trial targeting only acute staphylococcal PJI, 8 weeks of levofloxacin-rifampicin treatment was non-inferior to extended-duration treatment regimens (PP cure rates: 92% vs. 95%) [22]. Conversely, the multicenter randomized DATIPO trial, which included both acute and some early chronic cases, failed to demonstrate non-inferiority of a 6-week course compared to 12 weeks (failure rates of 30.7% vs. 14.5%, respectively, absolute difference 16.2%, 95% CI 2.9–29.5), thereby underscoring a twofold increased risk of treatment failure with the shorter duration [4]. It needs to be considered that definitions of acute, subacute, early chronic, and chronic inflammation are in large part empirical since they are based on the time at presentation. Even when periprosthetic soft tissue and bone specimens are histologically examined, the inflammatory infiltrate is variable due to tissue sampling at surgery and/or at pathological examination, and the pathologist’s interpretation.

Studies evaluating antibiotic duration after DAIR procedures have shown some methodological strengths but also significant limitations. Prospective cohorts like those by Berdal et al. [14], Soriano et al. [15], and Tornero et al. [19] offered standardized long-term follow-up in real-world settings, while studies such as Puhto et al. [20] and Lora-Tamayo et al. (2016) [22] included stratified analyses by duration with both ITT and PP populations, improving interpretability. Most notably, two randomized trials, Lora-Tamayo et al. (2016) [22] and Bernard et al. (2021, DATIPO) [4], provided the highest level of evidence on the topic [4,22].

Apart from these two trials, the overall quality remains limited due to the predominance of retrospective, single-center, and observational studies prone to confounding and indication bias. Treatment duration is often influenced by clinical factors, and antibiotic regimens vary widely in duration, route, and combination (especially rifampicin use), complicating comparisons. Definitions of outcomes are inconsistent, functional results are rarely reported, and microbiological or prognostic stratification, such as infection chronicity or host factors, is often lacking. Despite its significance, the DATIPO trial did not establish non-inferiority of the shorter duration regimen [4].

Nonetheless, based on the findings of the DATIPO trial, the most robust study to date on antibiotic duration in PJI, even though the DAIR subgroup analysis was not powered enough as a primary endpoint, we recommend against limiting antibiotic treatment to only 6 weeks in patients managed with DAIR, as this duration was significantly inferior to 12 weeks [4]. A discussion may be warranted as to whether intermediate durations, e.g., 8 or 10 weeks, as suggested by the trial conducted by Lora-Tamayo et al., could be sufficient to support a shorter standard duration than 12 weeks, while maintaining clinical efficacy and potentially offering the previously mentioned benefits [22].

There is an urgent need for a new well-powered randomized controlled trial specifically designed to evaluate optimal antibiotic duration in PJIs managed with DAIR. Such a study should incorporate stratification by key variables, including infection type (acute versus chronic), causative pathogens (with a focus on resistant organisms such as Methicillin resistant *Staphylococcus aureus* (MRSA) or Gram-negative bacilli), and relevant host factors (such as immune status, comorbidities, and implant type), in order to develop individualized, evidence-based treatment durations that balance efficacy with antimicrobial stewardship.

### 4.2. PJI Treated with One-Step Exchange Procedure (Table 3)

One-stage exchange is increasingly considered a viable alternative to the traditional two-stage approach for managing PJI, particularly in patients with favorable prognostic factors [24,25,26,27,28,29,30,31]. Compared to two-stage procedures, it offers several advantages, including reduced surgical workload, shorter hospital stays, quicker functional recovery, and lower overall costs [25,28]. Furthermore, the complete removal of the infected prosthesis may enhance infection clearance compared to DAIR. Although initially reserved for selected cases, one-stage exchange is now being used more broadly, including in patients with comorbidities or less favorable microbiological profiles [24,25]. According to IDSA guidelines published in 2013, a one-stage exchange should be followed by 2 to 6 weeks of pathogen-specific IV antimicrobial therapy, with an additional 3 months of oral therapy, based on limited evidence [6].

Indeed, several observational studies have investigated antibiotic treatment duration following one-stage exchange surgery for PJI, with significant variability in infection type (chronic versus acute), pathogen spectrum, and therapeutic strategies (see Table 3). Antibiotic regimens typically included 2 to 6 weeks of IV therapy [29,32,33], often followed by oral treatment for up to 3 months [25] or, in some cases, extended to 6 months [34]. Local antibiotic delivery, mainly gentamicin or vancomycin in cement or intra-articular administration, was frequently used as adjunctive therapy. Reinfection rates (RR) ranged from 0% [29] to over 24% [27,35], with lowest rates observed in studies involving early-stage or acute infections, and in those where local antimicrobial delivery was employed. Importantly, the type of pathogen emerged as a key determinant of outcome: in the series by Whiteside et al., patients with MRSA PJI were successfully treated with 2–4 weeks of IV therapy combined with 6 weeks of intra-articular vancomycin, achieving a RR of just 5.5% [32]. Conversely, Singer et al. reported a recurrence rate of 15% despite a 6-week rifampicin-fluoroquinolone regimen, with notably worse outcomes in patients with MRSA or MRSE (Methicillin resistant *Staphylococcus epidermidis*) infections and in those receiving hinged prostheses [26].

Studies including predominantly chronic infections reported success rates exceeding 85% with combined systemic and local therapy, provided that appropriate debridement and patient selection criteria were respected [24,36]. Tibrewal et al. showed excellent outcomes (RR 2%) in a cohort with chronic PJI managed with a 2-week IV and 3-month oral antibiotic strategy [33]. On the other hand, in cohorts including mixed acute and chronic infections without pathogen stratification or standardized protocols, RR exceeded 20% [27,35]. The inclusion of chronic infections associated with biofilm-producing organisms (e.g., coagulase-negative staphylococci or Enterobacterales) likely explains some of the poorer outcomes in these studies.

**Table 3:** Summary of Studies on Antibiotic Treatment Duration for Prosthetic Joint Infections managed by single-step exchange.

Study and Year (Ref.)	*n*/Design	Systemic Regimens (Major)	Systemic Exposure	Local/Suppression	Mean Follow-Up (Years)	Key Outcomes	Conclusion
Whiteside 2011 [32]	*n* = 18; retrospective cohort	Not reported	IV 2–4 weeks	Intra-articular vancomycin	5.1	Recurrence rate ~5.5%; KSS ~78 at 1 y → ~84–85 up to 6–8 y	Single-stage TKA for MRSA with 6 weeks intra-articular vancomycin controlled infection in most cases.
Singer 2012 [26]	*n* = 57; retrospective	Rifampicin + fluoroquinolone combos (~51%)	Total 6 weeks (2 w IV post-op → 4 w oral)	Local gentamicin	3	Recurrence ~15%; KSS ~72; function score ~71; Oxford-12 ~27	One-stage knee revision achieved high infection control when pathogen identified; outcomes worse with hinged prostheses and MRSA/MRSE.
Jenny 2013 [24]	*n* = 47; prospective observational cohort	IV vancomycin/teicoplanin; oral rifampicin + levofloxacin	IV 3.5 w (1–16); oral 12 w (3–16)	Long-term suppression: not reported	3	Recurrence ~12%; median pre-op KSS function 42; 56% had KSS > 150 post-op	Single-stage exchange is a viable alternative in chronic infected TKA, potentially reducing hospital burden and costs.
Baker 2013 [37]	*n* = 33; prospective	Not reported	Not reported	Not reported	0.6 (7 months)	Recurrence ~21%; OKS improved from 15 (95% CI 13–18) to 25 (95% CI 21–29)	No clear difference vs. two-stage; functional gains observed at short follow-up.
Shanmugasundaram 2014 [30]	*n* = 5; retrospective	Not reported	Not reported	Antibiotic spacers	2	Recurrence ~17%; other FO not reported	Initial success: hip PJI 1-stage 60% vs. 2-stage 70%; knee PJI 1-stage 80% vs. 2-stage 75%; better diagnostics needed.
Tibrewal 2014 [33]	*n* = 50; prospective	Not reported	IV 2 w → oral 3 mo	Antibiotic-impregnated cement	10	Recurrence ~2%; OKS from 14.5 → 34.5 at 1 y (Δ ≈ +20; *p* < 0.001)	Single-stage may match two-stage outcomes with lower cost and morbidity.
Cury R de PL 2015 [34]	*n* = 6; retrospective	Not reported	IV 2–4 w → oral 6 mo	Suppressive therapy in 4/6	3	Recurrence ~16.7%; WOMAC ~49.5	Reported success: DAIR 75%, one-stage 83%, two-stage 100% in small series.
Haddad 2015 [29]	*n* = 28; retrospective	Not reported	6 weeks (IV and/or oral)	Antibiotic-loaded cement (gentamicin, vancomycin)	2	Recurrence 0%; KSS higher in 1-stage vs. 2-stage (88 vs. 76; *p* < 0.001); pre-op KSS ~32	One-stage can be an alternative for selected chronic TKA infections; RCTs needed.
Zahar 2016 [36]	*n* = 46; retrospective	Not reported	IV 14.2 days (10–17)	Antibiotic-loaded cement (gentamicin, clindamycin, vancomycin)	10	Recurrence ~7%; HSS improved from 35 to 69.6	Overall infection control ~93% with favorable clinical recovery; further research warranted.
Cochran 2016 [35]	*n* = 3069; retrospective database	Not reported	Not reported	Not reported	6	Recurrence 24.6% at 1 y; 38.25% at 6 y	Two-stage reimplantation showed highest success despite ~19% recurrence; higher than single-stage and DAIR.
Jenny 2016 [25]	Intervention *n* = 54; control *n* = 77; retrospective case–control	Not reported	3 months	Not reported	2	Recurrence: 15% (intervention) vs. 22% (control); ~80% KSS > 160; no significant group difference	Patient selection did not markedly influence outcomes for single-stage exchange.
Massin 2016 [27]	*n* = 108; retrospective	Not reported	Not reported	Not reported	2	Recurrence 24%; IKS 88.6 ± 9.4	One-stage may be reasonable (e.g., in women) without increased recurrence; supports broader use in selected TKR.
Li 2018 [38]	*n* = 22; retrospective	Vancomycin	4–6 weeks	Not reported	5	Recurrence 9.1%; other FO not reported	No significant difference between 1- and 2-stage in satisfaction and infection control.
Castellani 2017 [28]	*n* = 14; retrospective	Not reported	Not reported	Not reported	1	Recurrence ~7.2%; FO not reported	Superiority of one- vs. two-stage and role of antibiotic-free intervals remain unclear; larger prospective RCTs needed.

Notes: Recurrence rate (RR) and functional outcomes (FO) follow original author definitions. Systemic exposure indicates total duration or IV → oral split where specified. RR: reinfection rate, FO: functional outcome, HSS: Hospital for Special Surgery knee score, IKS: International Knee Society Score, KSS: Knee Society Score, OKS: Oxford Knee Score, WOMAC: Western Ontario and McMaster Universities Osteoarthritis Index, TKR: total knee replacement, TKA: total knee arthroplasty, DAIR: debridement, antibiotics and implant retention, MRSA Methicillin-resistant Staphylococcus aureus, MRSE: Methicillin-resistant Staphylococcus epidermidis.

Several of these studies benefit from long-term follow-up, e.g., up to 10 years in Tibrewal et al. and Zahar et al., which strengthens the assessment of RR [33,36]. Some cohorts, such as Jenny et al. [24] and Haddad et al. [29], also prospectively evaluated both clinical and functional outcomes, offering a more comprehensive view of treatment success. However, major methodological limitations persist. Most studies are retrospective, single-centered, and lack randomization or control groups, leading to selection bias and confounding factors. Infection chronicity is often poorly defined, and few studies stratify by infection type or pathogen, despite their known prognostic impact. Antibiotic regimens are highly variable in both agents and duration, ranging from 2 weeks of IV therapy to 6 months of oral treatment, with limited reporting. Small sample sizes further reduce statistical power and generalizability [28,34]. Reinfection definitions are inconsistent, and functional outcomes are inconsistently reported.

Despite one-stage exchange being increasingly used in the management of PJI, not only in highly selected patients but also in broader clinical contexts [24,26,27,31], evidence guiding optimal antibiotic duration in this setting remains limited. To date, only the DATIPO trial has compared 6 versus 12 weeks of antibiotic therapy in the context of one-stage exchange, albeit in a subgroup analysis. The cure rate between groups (4.0% vs. 2.8%, respectively) was not statistically significant. Given the theoretical advantage of biofilm eradication following complete implant removal, shorter antibiotic courses (e.g., 6 weeks) may be sufficient in this setting [4]. However, a dedicated, adequately powered randomized controlled trial is urgently needed to validate this hypothesis and define evidence-based treatment durations for patients undergoing one-stage exchange.

### 4.3. PJI Treated with Two-Step Exchange Procedure (Table 4)

Two-stage exchange remains the most widely used surgical strategy for chronic PJI in Europe and North America and is still considered the gold standard in many settings [1,5,6]. This approach involves prosthesis removal, thorough debridement, placement of an antibiotic-loaded cement spacer, and subsequent reimplantation after a defined period of systemic antibiotic therapy. Despite its widespread use, there is no consensus on the optimal duration of antibiotic treatment in this context.

The IDSA recommends 4 to 6 weeks of pathogen-specific IV or highly bioavailable oral antimicrobial therapy for the medical management of PJI following resection arthroplasty, regardless of whether staged reimplantation is planned [6].

A number of observational and comparative studies have assessed the optimal duration of systemic antibiotic therapy in PJI managed by two-stage exchange, with significant heterogeneity in patient populations, microbiological etiologies, antibiotic strategies, and definitions of outcome (see Table 4).

Some early prospective single-center cohort studies, small in size and lacking control groups, focused on local antibiotic strategies and reported the use of very short systemic antibiotic regimens (ranging from 1 to 14 days), most often in combination with local antibiotic delivery systems such as vancomycin- or gentamicin-loaded cement [39,40,41,42].

**Table 4:** Summary of Studies on Antibiotic Treatment Duration for Prosthetic Joint Infections managed by two step exchange.

Study and Year (Ref.)	*n*/Site	Design	Dominant Organisms	Systemic Antibiotics (Major)	Systemic Exposure	Local Antibiotics	Mean Follow-Up (mo)	Key Outcomes (Additional Debridement/Reimplant Cultures+/Persistence–Relapse)	Conclusion
Taggart 2002 [40]	*n* = 33; hip and knee	Prospective observational; single center; non-comparative	93% Gram-positives; 71% staphylococci	Not reported	5 days	Vancomycin (local)	67	Add. debridement 0%; cultures+ at reimplant 9%; persistence/relapse 3%	Short systemic exposure with local vancomycin yielded low relapse but some positive reimplant cultures.
Hoad-Reddick 2005 [41]	*n* = 52; knee	Prospective observational; single center; non-comparative	63% staphylococci	None beyond prophylaxis (cefuroxime)	1 day	Various local agents	56	Add. debridement 12%; cultures+ 16%; persistence/relapse 9%	Minimal systemic therapy with local measures showed moderate culture positivity and relapse rates.
Hart and Jones 2006 [43]	*n* = 48; knee	Prospective observational; single center; non-comparative	96% Gram-positives; 76% staphylococci	Vancomycin	14 days	Vancomycin + gentamicin (local)	49	Add. debridement 13%; cultures+ 23%; persistence/relapse 13%	Two-stage with short systemic vancomycin and local antibiotics achieved acceptable but non-negligible failure.
Stockley 2008 [39]	*n* = 114; hip	Prospective observational; single center; non-comparative	61% staphylococci	None (cephalosporin prophylaxis)	1 day	Various local agents	74	Add. debridement 4%; cultures+ 16%; persistence/relapse 12%	Local strategies with minimal systemic therapy produced low reoperation but notable positive cultures.
Whittaker 2009 [42]	*n* = 44; hip	Prospective observational; single center; non-comparative	All Gram-positives; 72% staphylococci	Vancomycin	14 days	Vancomycin + gentamicin (local)	49	Add. debridement 7%; cultures+ 2%; persistence/relapse 7%	Short systemic vancomycin plus local therapy yielded low culture positivity and relapse.
McKenna 2009 [44]	*n* = 31; hip	Retrospective observational; single center; non-comparative	All Gram-positives; 77% staphylococci	Vancomycin	5 days	Various local agents	35	Add. debridement 0%; cultures+ 0%; persistence/relapse 0%	Very favorable outcomes reported despite brief systemic exposure.
Mittal 2007 [45]	*n* = 37; knee	Retrospective observational; multicenter; comparative (short vs. long IV)	MR staphylococci	Not reported	≥6 w IV vs. <6 w IV	Various local agents	51	Cultures+ 0%; persistence/relapse: short 13% (2/15) vs. long 9% (2/22); *p* = 0.07	Longer IV tended toward lower relapse, not statistically significant in small sample.
Hsieh 2009 [46]	*n* = 99; knee	Retrospective observational; single center; comparative	67% Gram-positives; 53% staphylococci	1st-gen cephalosporin + gentamicin	4–6 w vs. 7 d	Various local agents	43	Additional debridement: long 2/46 (4%) vs. short 1/53 (2%); persistence/relapse: long 4% vs. short 6%	No clear advantage of longer systemic therapy in this cohort.
El Helou 2011 [47]	*n* = 208; hip and knee	Retrospective observational; single center; comparative; propensity-adjusted	Mainly Gram-positives; 62% staphylococci	Not reported	4 w ± 7 d vs. 6 w ± 7 d	Vancomycin ± tobramycin (local)	60	Cultures+: short 6.1% vs. long 8.7%; persistence/relapse: short 16% vs. long 27%	Shorter systemic duration did not worsen outcomes after adjustment.
Benka-bouche 2019 [48]	*n* = 39; hip and knee	Single-center, open-label randomized clinical trial	Various	Vancomycin IV; oral fluoroquinolone	6 w (39–45 d) vs. 4 w (27–30 d)	Local tobramycin in 2 cases (5%)	26	No significant difference in PJI subgroup	Short (4 w) non-inferior to 6 w in small RCT subgroup.
Ma 2020 [49]	*n* = 64; knee	Retrospective observational; single center; comparative	69% staphylococci	Not reported	4–6 w vs. ≤7 d	Vancomycin ± aminoglycosides (local)	75	Need for salvage antimicrobials/surgery: long 26% (11/43) vs. short 14% (3/21)	Longer courses associated with fewer salvage events numerically.
Bernard 2021 [4]	*n* = 81; hip and knee	Multicenter, open-label randomized clinical trial	40% S. aureus	Rifampicin + fluoroquinolones (common)	6 w vs. 12 w	Not reported	≥24	Failure: 15% (6/40) vs. 5% (2/41); difference 10.1% (95% CI −0.9 to 22.2)	Signal favoring 12 w; authors recommend longer duration.

Notes: Systemic exposure is reported as total days/weeks where available. Local antibiotic use reflects agents applied at spacer or reimplantation. Outcome components are harmonized for comparability (additional debridement; positive cultures at reimplant; persistence/relapse). w: weeks.

These studies predominantly included chronic infections involving Gram-positive bacteria, with staphylococci accounting for 61% to 76% of isolates. Despite the short systemic exposure, reported rates of relapse or persistence were relatively low, ranging from 3% to 13%. For example, McKenna et al. [44] reported a 0% RR in a cohort of 31 patients (77% of staphylococci) receiving only 5 days of IV vancomycin combined with local antibiotics. In the study by Stockley et al. [39], involving 114 hip PJIs (61% of staphylococci), systemic antibiotics were limited to a single prophylactic dose, yet the persistence rate remained at 12%, and only 4% required additional debridement. These results suggest that, at least in selected chronic cases caused by susceptible Gram-positive organisms, aggressive surgical debridement and high local antibiotic concentrations may compensate for short-duration systemic treatment. Very short systemic antibiotic courses, even when combined with local antibiotic therapy, should not be recommended at this stage, given the paucity of supporting data and the methodological weaknesses of the available studies.

More recent retrospective comparative studies have attempted to define minimal effective durations. Mittal et al. [45] evaluated 37 knee PJIs due to methicillin-resistant staphylococci, comparing <6 weeks versus ≥6 weeks of IV antibiotics. The RR were similar (13% in the short group vs. 9% in the long group, *p* = 0.07), although the small sample size limits interpretability. Hsieh et al. [46] compared 7 days versus 4–6 weeks of IV cephalosporin plus gentamicin treatment in 99 knee PJIs (67% Gram-positive, 53% staphylococci), and found low RR in both groups (4% vs. 6%, respectively). El Helou et al. [47], in a larger retrospective cohort of 208 hip and knee PJIs (62% staphylococci), adjusted by propensity score, found that shortening systemic antibiotic therapy from 6 weeks to 4 weeks was associated with slightly higher RR (16% vs. 27%), though the difference was not statistically significant.

Two randomized controlled trials provide the most robust, albeit limited, evidence. Benkabouche et al. [48] randomized 39 PJI patients (hip and knee) to 4 versus 6 weeks of systemic antibiotics, primarily IV vancomycin followed by oral fluoroquinolones. Only two patients received local antibiotics (tobramycin), and no significant differences were observed in infection-free survival at 26 months. In contrast, the DATIPO trial (Bernard et al.) [4] enrolled 81 patients with mostly staphylococcal infections (40% *S. aureus*) and compared 6 versus 12 weeks of rifampicin-fluoroquinolone therapy. While the difference in failure rates was not statistically significant (15% for 6 weeks vs. 5% for 12 weeks; absolute difference 10.1%, 95% CI −0.9% to +22.2%), the data suggested a trend favoring prolonged therapy in this population, which included a proportion of late chronic infections. Importantly, neither trial stratified results by infection chronicity or by methicillin resistance, limiting conclusions for more complex or resistant pathogens.

Interestingly, in the DATIPO trial, the trend favoring 12 weeks of antibiotic therapy over 6 weeks appeared more pronounced in patients managed with two-stage exchange compared to those undergoing one-stage exchange [4]. This unexpected finding warrants confirmation through a dedicated trial specifically focused on the two-stage strategy. Such a study should also evaluate the optimal duration of antibiotic therapy following reimplantation, taking into account intraoperative microbiological findings at the time of the second-stage procedure.

### 4.4. PJI Treated with Total Removal Without Implantation

In certain clinical scenarios, particularly when reimplantation is not feasible or is deliberately deferred, definitive surgical strategies such as permanent resection arthroplasty, arthrodesis, or above-knee amputation may be considered. These options are typically reserved for patients with poor bone stock, extensive soft tissue damage, multiple prior treatment failures, or significant comorbidities, and they raise specific questions regarding the appropriate duration of antimicrobial therapy [1,5,6].

In PJI managed without reimplantation, antibiotic duration varies depending on the surgical approach and underlying infection burden. Very few data are available on this specific topic. In the setting of permanent resection arthroplasty, often selected for low-demand patients or those with contraindications to reconstruction, treatment regimens are highly variable [50,51,52]. In the cohort by Wasielewski et al., 82% of patients (41/50) received IV antibiotics for a median duration of approximately 6 weeks (±6 days), with oral suppressive therapy ranging from none to indefinite; the overall cure rate reached 94%, despite the absence of standardized oral continuation [52]. In cases managed by arthrodesis, typically indicated in younger or more active patients when reconstructive or two-stage procedures fail, parenteral antibiotic therapy is generally initiated perioperatively and continued orally for a total duration of 6 weeks, as described by Kutscha-Lissberg et al. [53]. Success rates for infection control and fusion range from 88% to 94% in small series [53,54,55]. In contrast, above-knee amputation, a salvage procedure reserved for life-threatening infections or non-reconstructable joint destruction, requires minimal antibiotic duration. According to the 2013 IDSA guidelines, if complete debridement is achieved and there is no residual infection (e.g., bacteremia or abscess), systemic antibiotics can be safely discontinued within 24–48 h postoperatively [6]. Across all three approaches, the absence of prosthesis reimplantation may theoretically allow for shorter antibiotic courses; however, the supporting evidence remains particularly limited and of low quality.

## 5. Discussion

In the context of PJI, treatment durations recommended in clinical practice and current guidelines often extend beyond three months, substantially longer than the six weeks typically advised for osteoarticular infections without prosthetic material [56]. This prolonged duration is primarily attributed to the challenges associated with eradicating biofilm-associated bacteria [57]. While several studies have investigated optimal treatment durations for PJI, the existing literature remains limited by various methodological and clinical constraints, despite the numerous potential benefits associated with shorter antibiotic regimens. Importantly, the OVIVA trial demonstrated that six weeks of oral antibiotics were not inferior to intravenous therapy for complex orthopedic infections, based on 1-year treatment failure [58].

From a microbiological standpoint, most studies involved predominantly Gram-positive infections (60–95%), with staphylococci representing the majority (often >70%). However, only a minority of studies explicitly reported rates of MRSA or coagulase-negative staphylococci, which are known to be more difficult to eradicate and more likely to relapse. Furthermore, the influence of Gram-negative or polymicrobial infections remains poorly characterized, as these pathogens were often excluded or underreported. Regarding infection chronicity, most studies implicitly targeted chronic PJI, given the two-stage protocol, but few explicitly differentiated chronic from acute hematogenous infections, or defined the duration of symptoms prior to intervention, which is a critical determinant of biofilm maturity and response to antibiotics.

Methodologically, while several studies used prospective designs and incorporated meaningful clinical and microbiological endpoints (e.g., intraoperative cultures at reimplantation, reoperation rates), substantial limitations persist. Most studies are single-center, non-randomized, and include relatively small sample sizes (often <100 patients). Definitions of relapse and treatment failure are heterogeneous, functional outcomes are inconsistently reported, and local antibiotic regimens vary widely or are incompletely described. Moreover, the lack of stratification by pathogen, antimicrobial susceptibility, and infection stage hinders the development of clear, evidence-based duration recommendations for different clinical scenarios.

To date, the DATIPO trial remains the only adequately powered randomized controlled trial available.

Indeed, Bernard et al. undertook a multi-center randomized controlled trial, in which they enrolled 410 patients suffering from hip or knee PJI. Participants were randomized to receive either 6 weeks or 12 weeks of antibiotic therapy, following surgical source control [4]. Patients were randomized with stratification according to the surgical approach. The primary outcome was infection persistence or recurrence within two years following cessation of antibiotic therapy, reported in 18.1% of the 6-week group vs. 9.4% of the 12-week group in the global population. The risk difference confidence interval precluded non-inferiority for the shorter course. Sub-group analysis revealed that inferior outcomes were accentuated in patients treated with DAIR, with an absolute risk difference of 16.2%. However, no significant differences in outcomes were observed between 6- and 12-week regimens in patients treated with either one- or two-stage revision. Although a trend favoring the 12-week duration was noted in the two-stage exchange group, these findings suggest that a 12-week antibiotic course may be necessary in cases involving retained infected hardware. Conversely, they also support the possibility of shorter treatment durations when PJI is managed with one- or two-stage exchange procedures [4].

However, in contrast to the DATIPO trial, a multicenter randomized controlled trial conducted in Spain by Lora-Tamayo and colleagues evaluated an 8-week antibiotic regimen compared to 3- and 6-month courses for the treatment of staphylococcal PJI of the hip or knee managed with DAIR [22]. The study found no significant differences in infection relapse rates between groups, suggesting that 8 weeks of treatment may be sufficient in this setting. These results question the systematic use of extended antibiotic durations [22]. The intention-to-treat analysis showed non-inferiority of clinical cure, but the trial was conducted using fluoroquinolone–rifampicin treatment groups, and excluded patients with poor initial prognosis or high risk of early treatment failure [30]. These findings provide a rationale for investigating whether 8 weeks of antibiotic therapy is non-inferior to 12 weeks.

Finally, due to DATIPO subgroup analysis, a trial specifically studying an antibiotic duration of 6 weeks during one step exchange is mandatory as single-stage revision surgery has gained increasing popularity in the management of PJI. Studies on single-stage revision have reported promising results, with RR ranging from 5% to 25% [24,26,27,28,29,30,32,33,34,35,36,37,38], which are comparable to those seen with two-stage revision procedures (9% to 20%) [39,40,41,42,43,44,45,46,47,48,49].

The results of the SOLARIO trial, a multicenter open-label randomized controlled non-inferiority study comparing short versus long systemic antibiotic therapy in conjunction with local antibiotic treatment for orthopedic infections, are highly awaited [59]. Participants will be randomized 1:1 to receive either a short course (≤7 days) or a standard long course (≥4 weeks) of systemic antibiotics. The primary endpoint is treatment failure within 12 months post-surgery, evaluated by an independent blinded Endpoint Committee. A non-inferiority margin of 10% will be applied in both per-protocol and intention-to-treat analyses. One potential limitation of this study is that it encompasses all bone and joint infections, which may reduce the specificity of the findings for particular subgroups, such as PJI [59]. The RandOmized Arthroplasty infection worlDwide Multidomain Adaptive Platform (ROADMAP) trial is a global, randomized, adaptive platform study designed to evaluate multiple surgical and antimicrobial strategies for prosthetic joint infection, with a primary focus on adult hip and knee arthroplasties. Its primary objective is to determine the effect of alternative, guideline-concordant interventions on 12-month treatment success through systematic randomization embedded in routine care. The platform structure enables concurrent comparisons, efficient integration of new interventions, and iterative dropping of ineffective options, providing robust, real-world evidence to improve outcomes for patients with PJI (https://www.roadmaptrial.com (accessed on 22 October 2025)).

While awaiting further data, this review highlights—based on the DATIPO trial—that 12 weeks of antibiotics are currently warranted for most PJIs [4].

## 6. Conclusions

This review underscores the complexity and variability of antibiotic therapy duration in bacterial PJI management, highlighting the need for a more standardized and evidence-based approach. While current practices often rely on prolonged courses, emerging data suggest that shorter durations may be effective in select cases, reducing the risks associated with extended antibiotic use. Future research should focus on pragmatic multicenter trials, to evaluate antibiotic treatment duration and antimicrobial-coated versus standard revision implants for prosthetic joint infection (hip/knee). The primary endpoint should be infection-related treatment failure at 24 months; key secondary outcomes include functional and quality-of-life measures, safety, health-economic endpoints, and resistance emergence. Allocation would be concealed (1:1), stratified by center, joint, surgical strategy, and chronicity, with blinded outcome adjudication and mixed-effects intention-to-treat analysis (Table A1).

Thus, the orthopedic and infectious disease communities can improve outcomes for patients with this challenging condition.

## Data Availability

No new data were created or analyzed in this study.

## Appendix A

**Table A1:** Standardized criteria for pragmatic multicenter trials in prosthetic joint infection (hip/knee), including endpoints and harmonized diagnostic/laboratory procedures across centers.

Domain	Minimum Specification (REQUIRED)	Preferred/Extended Specification	Notes and Quality Assurance
Population and Setting	Adults (≥18 y) with suspected or confirmed hip/knee PJI, managed by DAIR, one-stage, or two-stage revision; consent obtained.	Include pragmatic spectrum (acute hematogenous and chronic). Pre-register site capabilities and case-mix to ensure balance.	Use ICM 2018/MSIS criteria for screening; record referral pathway and symptom duration.
Case Definition	Uniform diagnostic criteria (ICM 2018). Require ≥2 concordant cultures OR sinus tract OR a major criterion.	Adjudication panel confirms case status blinded to allocation.	Provide pocket card/SOP; site initiation training; periodic source-data verification.
Randomization and Stratification	Central concealed allocation 1:1, stratified by center, joint (hip/knee), surgical strategy (DAIR/1-stage/2-stage), and chronicity.	Use permuted blocks with variable sizes; web-based IWRS.	Document concealment; monitor strata counts.
Interventions of Interest	A) Antibiotic duration strategy (e.g., 6–8 w vs. 12 w) OR B) antimicrobial-coated vs. standard revision implants.	Protocolized agent choices per organism; mandate rifampicin-based combos for staphylococci unless contraindicated.	Pre-specify dosing, IV-to-oral switch rules, interactions, adherence tracking.
Primary Endpoint	Infection-related treatment failure at 24 months (composite: persistent/recurrent PJI, unplanned reoperation for infection, infection-related death).	Time-to-event analysis with competing risks; blinded endpoint committee.	Endpoints charter; dual review with arbitration.
Key Secondary Endpoints	Function (Oxford Hip/Knee Score), QoL (EQ-5D-5L), safety (CTCAE), health-economics (LOS, readmissions, costs), resistance emergence (MDR colonization/infection; C. difficile).	PROMs at baseline, 3, 6, 12, 24 months; cost-utility (QALYs); microbiological cure at reimplantation for staged procedures.	Central training for PROMs; standardized AE coding; harmonized HE CRFs.
Follow-up Schedule	Discharge, 6 ± 2 w, 3, 6, 12, 24 months; phone backup allowed.	Extended 36–60 months registry add-on.	Missed visit policy; vital status via national registries where available.
Pre-analytical Specimen Handling	At surgery obtain ≥5 separate periprosthetic tissue samples; label site; sterile dry containers; deliver to lab ≤ 2 h (≤24 h if 4 °C).	Sonication of explanted components where available; inoculate one sample into blood-culture bottles at bedside.	Chain-of-custody forms; temperature/time stamps; deviation log.
Microbiological Culture (Analytical)	Aerobic/anaerobic media; tissues incubated ≥7 days; extend to 14 days for low-virulence organisms (e.g., Cutibacterium).	Quantitative sonicate-fluid cultures; standardized media panel across sites.	Inter-lab proficiency testing; document negative culture workflow.
Molecular and Biomarker Tests	PCR/16S optional but protocolized; synovial WBC and PMN% when feasible; CRP/ESR mandatory at baseline.	Central mNGS for discordant/negative cases (optional).	Report platform/version; validation required for non-standard assays.
Histopathology	PMN count thresholds documented a priori; frozen/permanent sections per local standard.	Central review of 10% random sample.	Standardized report template; slide digitization where feasible.
Susceptibility Testing	EUCAST (preferred) or CLSI breakpoints; method documented.	Central re-testing of sentinel isolates; synergy testing for rifampicin/fluoroquinolone when indicated.	Annual QC with reference strains; discrepancy reconciliation.
Data Elements (Minimum)	Demographics, comorbidities (CCI), prior antibiotics, symptom duration, joint, strategy, organism(s), MICs, implant details, spacer type, local antibiotics, IV/oral days, adherence, AEs, reoperations, PROMs, costs.	FAIR-mapped core outcome set; partial EHR import.	Locked data dictionary; SI units.
Analysis Plan	Mixed-effects ITT (center random intercept); multiplicity-controlled secondaries; predefined PP and as-treated analyses.	Bayesian hierarchical subgroup borrowing (organism, strategy).	SAP finalized before DB lock; independent statistical oversight.
International Harmonization	Translated SOPs; alignment with EU/Non-EU regulations.	Central kit provision (media, labels); remote monitoring.	Maintain a regulatory/ethics concordance matrix.
